# Feasibility and Acceptability of a mHealth Patient Navigation Intervention to Increase Pre-Exposure Prophylaxis Uptake in Racially and Ethnically Diverse Sexual and Gender Minority Youth in Los Angeles (PrEPresent): Pilot Randomized Controlled Trial

**DOI:** 10.2196/69255

**Published:** 2025-10-31

**Authors:** Sam Calvetti, Bryan Lei, Jacob B Stocks, Matthew T Rosso, Manuel Puentes, Ramon Durazo-Arvizu, Lindsay Slay, Michele D Kipke, Lisa B Hightow-Weidman

**Affiliations:** 1 Pediatric Research Administration, Children’s Hospital Los Angeles, 4650 Sunset Boulevard, Los Angeles, CA, 90027, United States, 1 3236602450; 2 Biostatistics and Data Management Core, The Saban Research Institute, Children's Hospital Los Angeles, Los Angeles, CA, United States; 3 Institute on Digital Health and Innovation, College of Nursing, Florida State University, Tallahassee, FL, United States; 4 Duke Clinical Research Institute, Duke University School of Medicine, Durham, NC, United States; 5 Department of Population and Public Health Sciences, Keck School of Medicine, University of Southern California, Los Angeles, CA, United States

**Keywords:** PrEP, mobile health, LGBTQ, adolescent, transgender, HIV, pre-exposure prophylaxis

## Abstract

**Background:**

Pre-exposure prophylaxis (PrEP) is a powerful tool to prevent the transmission of HIV. Interventions promoting PrEP must focus on populations most impacted by systemic barriers to uptake. Historically, young sexual minority men and transgender women have the highest demonstrated rates of new HIV diagnoses, but prevalence within other gender minority populations is now being studied. Few interventions have focused on addressing PrEP uptake with sexual and gender minority (SGM) youth, particularly through mobile health (mHealth) technologies. Built on the successful foundation of the HealthMpowerment Platform, PrEPresent aimed to engage SGM youth across diverse gender, racial, and ethnic identities in the Greater Los Angeles area.

**Objective:**

The aim of this study was to evaluate the feasibility and acceptability of a digital peer patient-navigation PrEP uptake app.

**Methods:**

PrEPresent incorporated patient activation theory into an mHealth intervention. The study took place over a 6-month period with visits at baseline, 3 months, and 6 months. The intervention period lasted from baseline to 3 months. Control participants received an information-only app. Intervention participants received an enhanced app and access to an interventionist, the PrEPresentative. Intervention participants could meet with the PrEPresentative four times over the 3-month period via phone, Health Insurance Portability and Accountability Act–compliant videoconferencing, or an in-app text messaging. PrEP uptake was measured through survey responses and the UrSure rapid urine test of tenofovir.

**Results:**

PrEPresent comprised of 147 sexual and gender-diverse participants—75 participants were randomized into the control arm and 72 into the intervention arm. A total of 48% (71/147) were Latinx and 18% (27/147) were Black or African American. Most (98/147, 67%) were transgender or gender diverse, and the remaining (49/147, 33%) were cisgender men. PrEP was initiated by 25% (14/56) of intervention participants and 19% (11/58) of control participants. In total, 50% (36/72) of intervention participants completed two or more sessions with the interventionist. Intervention participants had an average of 15.93 (SD 15.85) logins compared to 6.31 (SD 9.27) logins for control participants. Average use of the mHealth platform was 9.51 (SD 11.47) minutes for intervention participants and 3.03 (SD 5.70) minutes for control participants.

**Conclusions:**

PrEPresent met primary outcome measures of feasibility and acceptability. Despite this, PrEP uptake was low, and use of the platform was low compared to other HealthMpowerment projects. While mHealth offers promising HIV prevention outcomes, fostering active app engagement is crucial in promoting behavior change. Mixed success in initiating PrEP uptake across mHealth interventions involving SGM youth warrants further inquiry into how these platforms can address prevention barriers with this population. Interventions targeting uptake and adherence will need to adapt as the landscape of PrEP delivery evolves with the adoption of on-demand and long-acting injectable modalities.

## Introduction

### Background

Despite a decrease in new HIV acquisitions within cisgender men (those who identify their gender with their sex assigned at birth) in the LGBTQ+ community, 67% of new HIV acquisitions are still attributed to sexual interactions within this group. Young sexual minority men, those between the ages of 13 and 34, make up just over half (56%) of new diagnoses [1]. Historical erasure of expansive gender identities has contributed to a lack of understanding of HIV likelihood and prevalence within transgender and nonbinary people (those who identify their gender differently from their sex assigned at birth) [2]. The most well-documented incidences are in transgender women, who have a higher rate of HIV acquisition than any other population subgroup. One count within major US cities in a 2019‐2020 Center for Disease Control Special Report cited a 42% positivity rate within this population, with 18.9% of the sample living with HIV aged 18‐29 years [3]. HIV prevention strategies must address the needs of populations like young sexual minority men and young transgender women while working to prioritize the inclusion of all sexual and gender minority (SGM) youth.

While similar HIV prevention issues have been noted for both young sexual minority men and gender minority youth populations, each group has unique needs to overcome barriers to care [4,5]. The intersection of marginalized identities can differently impact their ability to engage in the HIV care continuum [6]. Individual level issues (internalized stigmas, isolation, mental health, or substance use), social level issues (stigmas perpetuated by social relations through friends, family, or relationships), and institutional level issues (transphobia, homophobia, and racism) create health disparities among SGM people that impact their ability to access and continually engage in HIV prevention and treatment [7,8]. More research is needed to understand the individualized needs of SGM youth. Understanding overlapping needs could tailor engagement strategies to best engage people with SGM identities.

### Pre-Exposure Prophylaxis Awareness, Utilization, and Uptake-Advancements in Pre-Exposure Prophylaxis

An important factor to end the HIV epidemic is increasing the uptake of pre-exposure prophylaxis (PrEP), a medication that can prevent the transmission of HIV [9]. In the United States, PrEP awareness among SGM youth continues to be high, but uptake is low [10,11]. More research is needed to understand how these factors impact SGM populations. Low uptake in this group has been attributed to different factors, such as a lack of trust in the medical system by transgender people interested in PrEP [12] and social stigma around PrEP disclosure due to negative connotations of PrEP users among Black SMM communities [13]. In a 2021 review of interventions targeting PrEP uptake, adherence, or persistence in the United States, 20 published interventions were identified, but only two listed uptake as an intended impact [14]. Additional studies are necessary to understand how interventions can increase this health behavior, particularly in SGM youth.

Not all forms or methods of PrEP are approved to be used by all people. The current approved forms of PrEP for people assigned female at birth who have receptive vaginal sex are daily oral Emtricitabine/Tenofovir Disoproxil Fumarate and Cabotegravir injectable [15]. Injectable PrEP was approved to be used in December 2021 [16]. Emtricitabine/Tenofovir Alafenamide (TAF), an alternative daily medication approved for PrEP in October 2019, is only for use in people assigned male at birth [17,18]. Although not US Food and Drug Administration–approved, guidance exists for on-demand PrEP (also called 2-1-1), but its efficacy has only been well-studied in gay and bisexual cisgender men [19]. Patient navigation, empowerment, and education around uptake are essential within SGM youth because of the availability and accessibility of different types of PrEP for different types of people.

### Mobile Health Interventions

Mobile health (mHealth) interventions can play a key role in mitigating factors that contribute to HIV acquisition among SGM youth in the United States. These interventions have been shown to be acceptable in many contexts: in a national sample of PrEP adherence with a majority population of young sexual minority men of color [20], by increasing self-HIV testing within a cohort of 202 transgender youth recruited digitally [21], and in access to HIV/sexually transmitted infection (STI) at home testing kits, condoms, lube, and prevention information among transmasculine people living in Atlanta, Georgia, and Washington, DC [22]. mHealth has impacted HIV-related stigma in both people living with HIV and those not living with HIV [23]. There is a lack of information about how mHealth solutions can affect the uptake of PrEP, particularly in populations including both young sexual minority men and transgender and nonbinary people. PrEPresent, a mHealth app built on the successful foundation of the HealthMpowerment (HMP) Platform [24], prioritized PrEP uptake as a health behavior change and aimed to engage with SGM youth across diverse gender and racial and ethnic identities in the Greater Los Angeles area.

### Project Aims

A pilot randomized control trial (RCT; ClinicalTrials.gov NCT05281393) was conducted to evaluate a tailored mHealth intervention, PrEPresent, which sought to increase PrEP uptake among SGM youth at a greater likelihood of HIV acquisition currently outside of the care continuum. PrEPresent connected users with a mobile app and a trained intervention peer support navigator, the PrEPresentative, and provided educational and motivational support to SGM youth in their decision to initiate PrEP. Information about the session content and other components of the intervention is outlined in the research protocol [25]. PrEPresent incorporated patient activation theory, which focuses on one’s ability to manage their own wellness and health care [26]. The aim of this project was to evaluate the feasibility and acceptability of the app and intervention with a racially and ethnically diverse sample of 150 SGM youth (aged 16‐26) in Los Angeles.

## Methods

### Study Design and Population

Formative research was first conducted to inform app features, content, and resources (Phase 1A), including 13 qualitative interviews with key informants providing PrEP care in the Los Angeles area. These interviews were transcribed and reviewed by a coding team of two infectious disease physicians. Overarching themes from the interviews were: location of services or descriptions of clinics, description of PrEP-specific provider training, basic provider duties around PrEP uptake, adherence, and persistence, source of clinic referrals, client motivators and facilitators, client barriers, clinical motivators, PrEP care specific to transgender populations, conversations about PrEP or sexual health, PrEP prescribing practices, client and provider stigma, COVID-19 impacts, and app suggestions or resources. These interview findings informed the formation of working groups and contributed to the addition of Los Angeles–specific resources and studies to the app (Phase 1B). The working groups included four weekly Zoom (Zoom Video Communications, Inc) sessions with 20 SGM youth with a goal of understanding PrEP awareness, acceptance, readiness, and hesitation. Each group consisted of 10 SGM youths who met study criteria; the working group sessions were facilitated by a member of our research team. The four sessions covered: health and sexuality, PrEP, mHealth apps, and a guided feedback session for the proposed mHealth for PrEP app. Working group findings were summarized into an information packet. This was shared with app developers to inform app tailoring to youth preferences, including the desire for a rewards system, varied avatar representations, and the general structure of the app’s design. Youth Advisory Board (YAB) members also reviewed these findings to inform SGM youth–specific study and resource creation. The overall takeaway from the youth working groups was PrEP information sources, barriers and activators to accessing health care and PrEP, experiences in STI or HIV testing, prescription or adherence concerns, and familiarity with PrEP navigators. Phase 2 involved preliminary usability testing of the PrEPresent platform with 7 SGM youths, and Phase 3 involved 1:1 randomization of racially and ethnically diverse SGM youth into a pilot RCT.

In Phase 3, participants completed study visits at baseline, 3 months, and 6 months. The intervention was delivered between the baseline and 3-month visits. Most study activities took place digitally, but participants could elect to complete procedures in person. Participants completed computer-assisted self-interviewing via a web-based survey at baseline. At the 3- and 6-month time points, all participants completed computer-assisted self-interviewing surveys and a biomarker test of PrEP adherence, the UrSure point-of-care rapid urine test of tenofovir. UrSure is a validated urine assay with high sensitivity and specificity for tenofovir use within the previous 48 hours [27]. UrSure test kits were either mailed to participants or completed on-site at the research location. If all study components were completed (three surveys and two UrSure biomarker tests), participants received a bonus incentive.

A total of 147 participants were recruited into the study between August 2022 and July 2023. Participants were recruited using a variety of strategies, including recruitment from an existing cohort of young sexual minority men and transgender and nonbinary youth of color in Los Angeles [28], distribution of fliers at community-based organizations and tabling at community events, and through web-based advertising via social media and dating apps (eg, Instagram, Facebook, and Jack’d). Eligibility criteria for participants were as follows: (1) aged 16‐26 years; (2) cisgender men, transgender or gender nonconforming people, or identified differently from the gender picked for them at birth; (3) gay, bisexual, or some other same-sex identity, or reported having had sex with anyone with a penis during the previous 12 months; (4) White or Caucasian, Black or African American, Hispanic or Latino or Latinx, or multiracial with one of these identities; (5) living in the Los Angeles metro area; (6) had daily access to an iOS or Android smartphone or tablet with internet access; (7) reported having insertive or receptive sex in the previous 6 months or reported a positive STI result in the previous 6 months; (8) not currently on PrEP and had no plan to start or restart PrEP in the following 7 days; and (9) not currently enrolled in another HIV prevention study. Participants who participated in Phases 1 and 2 of the research were not eligible to participate in Phase 3. Individuals were not eligible to participate if they were living with HIV or were non-English speaking. Contact information for potential participants, such as name, phone number, email address, and date of birth, was stored in a recruitment database. This information was used to ensure participants could only enroll once in the project.

### Randomization Mechanism

A Microsoft Excel–based simple random assignment approach was used for the PrEPresent Study. The RANDBETWEEN function with a range of 0 to 1 was used to generate a list of 150 continuous random numbers. Randomly generated numbers with a value greater than or equal to 0.5 were denoted as intervention allocations, while numbers with a value less than 0.5 were denoted as control allocations. This resulted in a 1:1 list of 75 assignment allocations per study arm. No stratification or blocking was used to generate the random assignments. The study data manager generated the assignment list independently of other study team members. All study personnel beyond the data manager were blinded to the order of assignments prior to individual participant randomization. Upon completion of consent and the baseline survey, study research assistants used the participant management system to prospectively assign participants to intervention or control, at which point they became aware of their assignment. From there, participant arm assignment was unblinded.

### Study Procedures

#### Overview

Following verification of eligibility, participants completed in-person or virtual electronic consent processes via videoconferencing, then performed a baseline survey. After survey completion, participants were randomized into the intervention (PrEPresent app) or control (information-only app) arm and were prompted to download the study app. Baseline visits concluded with a tour of the app and its features. Participants were encouraged to use the app daily, and SMS text message push reminders were sent to those who had not logged into the app for 7, 14, 30, or 60 days.

#### Intervention Arm

Intervention participants were encouraged to schedule their first session with the PrEPresentative, the interventionist peer support navigator. Participants received SMS text message reminders for their confirmed appointments 24 hours prior and 1 hour prior. Participants were encouraged to meet with the interventionist monthly, with weekly check-ins occurring via text or phone call between sessions. The PrEPresentative was trained on an intervention curriculum. The curriculum included guided session topics, a reference library, motivational messaging examples, common solutions to known PrEP barriers, and additional information about PrEP. Sessions were conducted via an in-app chat feature, phone call, or Health Insurance Portability and Accountability Act–compliant video call. Each intervention session had to be completed before moving on to the next. Following intervention sessions, the PrEPresentative completed a case report form to track intervention statistics. Session content and referrals were recorded, and multiple session topics could be chosen. Session topics were broken into three overarching categories: individual, sociocultural, and structural. After each session, participants were sent a brief satisfaction survey. Intervention participants had full access to the PrEPresent app. App features included a health tracker for habit monitoring, an anonymous user forum, activities and goal-setting functions, avatar customization, achievement badges, a care locator, an ask the expert section, and an interventionist appointment scheduler.

#### Control Arm

Control participants were provided with an information-only version of the app. This app contained a media library with resources regarding PrEP and did not allow access to other features. Control participants could not interact with the PrEPresentative.

### Outcomes

During this initial work to evaluate the PrEPresent intervention, the primary outcomes were acceptability and feasibility. Outcomes were prespecified in the protocol and trial registration as being assessed at the 3-month follow-up. This time point was selected based on the anticipated window of initial behavior change following exposure to the digital intervention. We defined acceptability as participants’ use of the app at higher rates than the control arm. Feasibility, in turn, was defined as at least 50% of intervention participants completing two peer navigation sessions with the PrEPresentative. A brief satisfaction assessment was administered at the end of each navigation session to identify challenges experienced by the participant, the most and least helpful aspects of the session, challenges using the system, satisfaction with information provided, and any additional needs the navigator did not address. The intervention sessions were defined as feasible if an overall postsession score of 4 or higher (out of 7) was reported on these surveys. Metrics on feedback about components of the app, such as trustworthiness of information, ease of use, and future app use, were gathered to understand app acceptance. The secondary outcome measure was defined as higher levels of PrEP uptake within intervention participants compared to control participants. PrEP uptake was defined as an adherent UrSure test result or self-report PrEP use on survey measures.

### Statistical Analysis

All statistical analysis was performed using R (version 4.2.3, R Core Team). Descriptive statistics were calculated and stratified by treatment arm. Continuous variables were summarized using medians and IQR. Categorical variables were reported using frequencies and percentages. The Fisher exact test and the Wilcoxon rank sum test were used to compare variables of interest between arms. Cronbach α was calculated to assess the internal consistency of nonvalidated instruments. The total scores of these instruments were calculated by summing the individual question scores. Due to the descriptive nature of our pilot trial, statistical analysis did not include power calculations or tests of statistical significance. Instead, 95% CIs are provided when applicable.

### Ethical Considerations

All phases of PrEPresent were approved by the Children’s Hospital Los Angeles Institutional Review Board (CHLA-20‐00596) under expedited review. The study involved three phases of research, as mentioned above. For Phase 1A, a waiver of signed informed consent was approved. Participants received an information sheet, and verbal consent was obtained. For Phases 1B, 2, and 3, participants signed informed consent and Health Insurance Portability and Accountability Act authorization documents. A waiver of permission for parental consent was obtained for minor participants due to the sensitive nature of the study eligibility criteria, in line with California-based protections for minors accessing sexual health services. To maintain patient privacy and confidentiality, only minimal identifying information necessary to maintain contact with participants during study procedures was collected and stored in a password-protected database. All study data were deidentified and monitored under a pre-established data safety and monitoring plan. Participants were prompted to use an anonymous username to enroll in the app and were discouraged from posting identifying information. Participants were compensated via Cash App, PayPal, Venmo, or Visa e-gift cards. In Phase 1A, PrEP care providers and PrEP navigators participating in qualitative interviews were compensated US $35 for their participation in a 60-minute interview. In Phase 1B, SGM youth participants received US $50 per working group session for 4 sessions, with a chance to receive an additional US $50 bonus for up to a total of US $250. In Phase 2, SGM youth participants pilot-testing the app received US $40 for the pretest survey measure and focus group attendance and US $40 for a posttest survey measure and one-on-one interview participation for a total of US $80. In Phase 3, pilot RCT participants received US $50 for completing each survey and US $20 for biomarker test completion. If participants successfully completed all study activities, they received an additional US $50 bonus. Intervention arm participants earned US $10 for completing each postsession survey. Participants in the intervention arm could earn up to US $280, and participants in the control arm could earn up to US $240.

## Results

### Demographics and Comparisons

**Figure 1.**
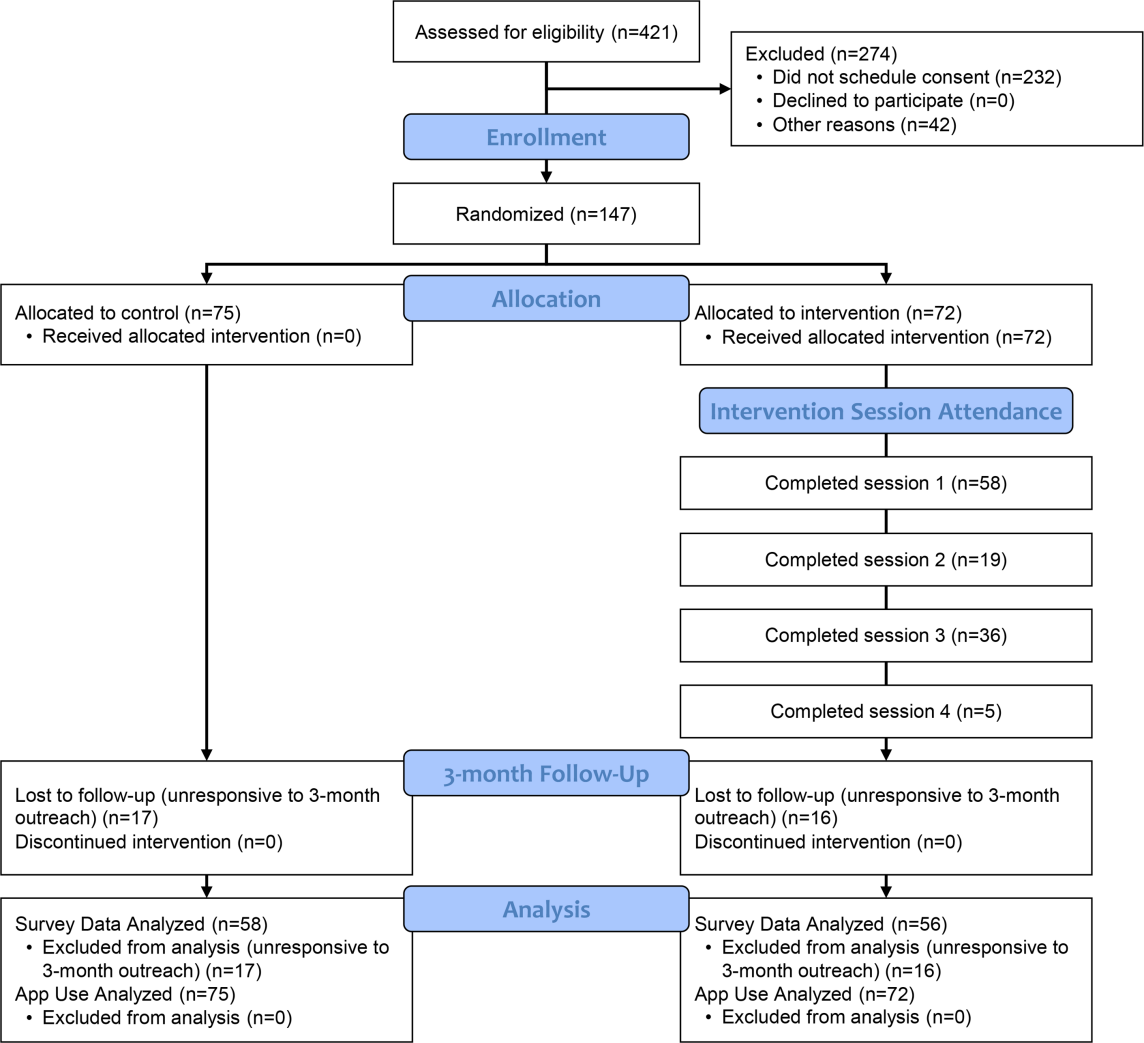
PrEPresent CONSORT flow diagram. CONSORT: Consolidated Standards of Reporting Trials.

Of those determined eligible via digital screener, 147 participants were enrolled in the study, and 274 participants were excluded. Most (n=232) were excluded due to not confirming eligibility and scheduling an initial consent visit. A total of 42 participants confirmed their eligibility but did not attend a consent visit due to other reasons. Of the 147 participants enrolled in the study, 72 participants were randomly assigned to the intervention arm and 75 to the control arm (Figure 1). The mean age for the sample was 22.57 (SD 2.48) years. Gender was measured using a two-part question assessing sex assigned at birth and gender identity, with gender being measured with a “select all that apply” approach that also allowed for a write-in. If participants selected more than one gender, they were prompted to select which gender they most identified with. These responses were collapsed, as presented in Table 1. Gender varied greatly among participants, with 33% (47/147) of the participants identifying as cisgender men, followed by nonbinary individuals (42/147, 29%), and trans men (19/147, 13%). Gay (41/147, 28%) and queer (39/147, 27%) were the most common sexual orientations, making up more than 50% (80/147) of the participants. Almost 62% (91/147) of participants reported a sexual attraction to more than one gender. Hispanic or Latinx was the most represented group (71/147, 48%), predominantly in the control arm (42/75, 56%) compared to the intervention arm (29/72, 40%). Black or African American-identified participants made up 21% (15/72) and 16% (12/75) of the intervention and control arms, respectively. A detailed breakdown of the demographic characteristics is summarized in Table 1.

**Table 1.** Baseline demographic characteristics of sexual and gender minority youth in the PrEPresent study^a^^,b^.

Characteristic	Total (N=147)	Control (n=75)	Intervention (n=72)
Age (in years), mean (SD)	22.57 (2.48)	22.55 (2.52)	22.60 (2.45)
Sex at birth^a^, n (%)			
Male	78 (53)	40 (53)	38 (53)
Female	66 (45)	32 (43)	34 (47)
Intersex	1 (1)	1 (1)	0 (0)
Gender, n (%)			
Cisgender man	49 (33)	26 (35)	23 (32)
Gender nonconforming	6 (4)	0 (0)	6 (8)
Genderfluid	8 (5)	4 (5)	4 (6)
Genderqueer	6 (4)	5 (7)	1 (1)
Nonbinary	42 (29)	20 (27)	22 (31)
Trans woman	10 (7)	5 (7)	5 (7)
Trans man	19 (13)	10 (13)	9 (13)
Two-spirit	1 (1)	1 (1)	0 (0)
Other	6 (4)	4 (5)	2 (3)
Sexuality, n (%)^b^			
Bisexual	28 (19)	10 (13)	18 (25)
Demisexual	1 (1)	0 (0)	1 (1)
Gay	41 (28)	21 (28)	20 (28)
Heterosexual (straight)	1 (1)	0 (0)	1 (1)
Homosexual	3 (2)	2 (3)	1 (1)
Lesbian	3 (2)	3 (4)	0 (0)
Men who have sex with other men	2 (1)	1 (1)	1 (1)
Pansexual	24 (16)	13 (17)	11 (15)
Queer	39 (27)	22 (29)	17 (24)
Same gender-loving	1 (1)	1 (1)	0 (0)
Other	3 (2)	2 (3)	1 (1)
Race or ethnicity			
Black or African American	27 (18)	12 (16)	15 (21)
Hispanic or Latinx	71 (48)	42 (56)	29 (40)
White	49 (33)	21 (28)	28 (39)

a 2 “Decline to answer” in control.

b 1 “Don’t know” in intervention.

### Patient Activation

The Patient Activation Measure was used, with a Cronbach α of 0.93, to measure participants’ activation at each survey timepoint [29]. Both study arms scored within the high-level activation category (category 4, defined as 67.1‐100 points) at baseline. Patient activation scores remained stable across both arms over the 3 months (Table 2).

**Table 2.** PrEP^a^ eligibility, readiness, knowledge, and uptake among participants from baseline to 3 months.

Variables	Baseline control (n=75)	Baseline intervention (n=72)	3-month control (n=58)	3-month intervention (n=56)
PrEP readiness ruler				
On a scale from 1 to 10, how important is starting PrEP to you right now?, n (%)				
1‐5	51 (68)	50 (69)	—^b^	—
6‐10	24 (32)	22 (31)	—	—
On a scale from 1 to 10, how motivated do you feel to start PrEP right now?, n (%)				
1‐5	48 (64)	50 (69)	—	—
6‐10	27 (36)	22 (31)	—	—
PrEP Knowledge				
Before today, what types of PrEP had you heard about? (check all that apply), n (%)				
Oral PrEP	65 (87)	62 (86)	—	—
On-demand PrEP (2-1-1)	24 (32)	28 (39)	—	—
Injectable	21 (28)	15 (21)	—	—
Other	1 (1)	0 (0)	—	—
I have never heard of PrEP	7 (9)	7 (10)	—	—
Where did you hear about or get information on how to use PrEP in the last 6 months?^c^, n (%)				
Pharmacist	3 (4)	2 (3)	—	—
Doctor	24 (35)	24 (37)	—	—
Friends	26 (38)	38 (59)	—	—
HIV counselor	12 (18)	20 (31)	—	—
Social media	26 (38)	20 (31)	—	—
Other	15 (22)	11 (17)	—	—
Patient Activation Score				
Mean (SD)	78.02 (16.61)	77.74 (20.55)	77.06 (18.79)	80.13 (16.54)
PrEP uptake, n (%)				
Prebaseline PrEP uptake (lifetime)	14 (19)	16 (22)	—	—
PrEP uptake (3 mo)	—	—	11 (19)	14 (25)
PrEP type (select all that apply)^d^				
Daily oral pill	14 (100)	15 (88)	7 (64)	14 (100)
Injectable PrEP	0 (0)	1 (6)	1 (9)	0 (0)
On-demand PrEP (2-1-1)	0 (0)	1 (6)	0 (0)	0 (0)
Undetermined^e^	—	—	3 (27)	—
PrEP UrSure Adherence Test results^f^				
Adherent	—	—	5 (14)	4 (12)
Nonadherent	—	—	28 (80)	30 (88)
Intermediate	—	—	2 (6)	0 (0)
UrSure adherence among PrEP users^g^				
Adherent	—	—	5 (56)	4 (40)
Nonadherent	—	—	4 (44)	6 (60)

a PrEP: pre-exposure prophylaxis.

b Data not collected.

c n=68 for control, n=65 for intervention due to skip logic.

d n=14 for baseline control; n=17 for baseline intervention; n=11 for 3-month control; and n=14 3-month intervention.

e Undetermined participants tested adherent on UrSure Biomarker but did not report PrEP use at 3 months

f n=35 for 3-month control; and n=34 for 3-month intervention.

g n=9 for 3-month control; and n=10 for 3-month intervention.

### PrEP Readiness, Knowledge, Uptake, and Adherence

Only 14 participants reported not having heard of PrEP before participating in this study (7 in the intervention and 7 in the control arm). Although most participants had heard about oral PrEP (62/72, 86% and 65/75, 87% in the intervention and control arms, respectively), there was less overall knowledge of on-demand or injectable PrEP indications. Both arms had similar rates of lifetime prior PrEP use, with 22% (16/72) of intervention and 19% (14/75) of control arm participants reporting prior use. Participants were most likely to have heard about PrEP from their friends (64/147, 44%), a doctor (48/147, 33%), social media (46/147, 31%), or an HIV counselor (32/147, 22%). In an assessment of PrEP readiness, almost 70% (101/147) of both study arms reported the importance of starting PrEP between 1 and 5 on a 10-point scale. A total of 69% (50/72) of intervention participants and 68% (51/75) of control participants rated their motivation to start PrEP as between 1 and 5 on a 10-point scale. PrEP uptake was initiated by 14 (25%) intervention participants at 3 months and 11 (19%) participants in the control arm. Nearly all participants reported using daily oral PrEP. However, one control participant reported using injectable PrEP. Information on PrEP type is missing for three control participants, as they tested adherent on UrSure but did not report PrEP use in their 3-month assessment.

PrEP biomarker testing had a low rate of return across both arms. At the 3-month assessment, only 34 of the 56 (61%) intervention participants and 35 of the 58 (60%) control participants had completed their testing. Reported adherence levels according to tests were low across the sample, with 88% (30/34) of intervention participants and 80% (28/35) of control participants demonstrating nonadherence based on test results at 3 months. Of the 14 intervention participants who reported PrEP uptake, 10 completed biomarker testing and 4 (40%) were found to be adherent, compared to 11 control participants, of whom 9 completed biomarker testing and 5 (56%) were found to be adherent.

### Intervention

#### Session Content and Information

Intervention acceptability and feasibility are summarized in Table 3, including session delivery method, topic coverage, participant referrals, session satisfaction, and app satisfaction. In total, 58 (81%) participants attended the first session, 36 (50%) participants attended 2 sessions, 19 (26%) attended three sessions, and 5 (7%) attended four sessions. Phone call and in-app message-based intervention sessions were similarly used (50/118, 42% and 49/118, 42%, respectively), but most sessions took place outside of the app (video or phone call; 69/118, 59%). Motivation was discussed in 88% (104/118) of sessions, with PrEP knowledge as the second most discussed topic. HIV knowledge was discussed in 20% (23/118) of sessions, with sexual health being discussed in 35% (41/118). Referrals were provided to participants in 34% (40/118) of sessions. The most common referrals were to PrEP clinics, with the second to PrEP educational materials. Nine referrals to PrEP navigators were made.

**Table 3.** Session attendance, delivery, acceptability, and feasibility of the PrEPresent intervention (n=72).

Variables	Total sessions (n=118)	Session 1 (n=58)	Session 2 (n=36)	Session 3 (n=19)	Session 4 (n=5)
Attendance, n (%)	118 (100)	58 (81)	36 (50)	19 (26)	5 (7)
Session delivery, n (%)					
Video call	19 (16)	10 (17)	4 (11)	3 (16)	2 (40)
In-app messaging	49 (42)	20 (35)	17 (47)	9 (47)	3 (60)
Phone call	50 (42)	28 (48)	15 (42)	7 (37)	0 (0)
Session topics^a^, n (%)					
Individual					
Motivation	104 (88)	53 (91)	32 (89)	15 (79)	4 (80)
Adherence	12 (10)	2 (3)	5 (14)	3 (16)	2 (40)
Sexual health	41 (35)	28 (48)	7 (19)	5 (26)	1 (20)
Self-efficacy	24 (20)	13 (22)	6 (17)	4 (21)	1 (20)
Patient activation	49 (42)	26 (45)	13 (36)	9 (47)	1 (20)
PrEP^b^ Knowledge	66 (56)	56 (97)	8 (22)	1 (5)	1 (20)
HIV Knowledge	23 (20)	17 (29)	4 (11)	1 (5)	1 (20)
Other	55 (47)	26 (45)	16 (44)	10 (53)	3 (60)
Sociocultural					
Relationships	15 (13)	10 (17)	4 (11)	1 (5)	0 (0)
Other	4 (3)	2 (3)	1 (3)	1 (5)	0 (0)
Structural					
Stigma	2 (2)	2 (3)	0 (0)	0 (0)	0 (0)
Insurance	19 (16)	14 (24)	4 (11)	1 (5)	0 (0)
Work or employment	3 (3)	0 (0)	1 (3)	2 (11)	0 (0)
Other	5 (4)	2 (3)	1 (3)	2 (11)	0 (0)
Referrals^a^^,^^c^	40 (34)	24 (41)	13 (36)	3 (16)	0 (0)
PrEP navigator	9 (8)	6 (10)	3 (8)	0 (0)	0 (0)
PrEP clinic	21 (18)	15 (26)	8 (22)	1 (5)	0 (0)
PrEP educational materials	13 (11)	8 (14)	4 (11)	1 (5)	0 (0)
Sexual health resource	1 (1)	1 (2)	0 (0)	0 (0)	0 (0)
STI^d^ or HIV testing	4 (3)	2 (3)	1 (3)	1 (5)	0 (0)
Community clinic	1 (1)	0 (0)	1 (3)	0 (0)	0 (0)
Postintervention session satisfaction					
Mean (SD)	6.23 (1.09)	6.23 (1.01)	6.25 (1.15)	6.15 (1.20)	6.35 (1.04)
PrEPresent Care Navigator Satisfaction (n=55), n (%)					
Very satisfied	31 (56)	—^e^	—	—	—
Mostly satisfied	16 (29)	—	—	—	—
Indifferent or mildly dissatisfied	5 (9)	—	—	—	—
I did not talk to the care navigator	3 (6)	—	—	—	—

a Substance use, transportation, food insecurity, and housing were either not discussed or discussed across only one session.

b PrEP: pre-exposure prophylaxis.

c No referrals were made to the following: PrEP Income Support, PEP, Gender Affirming Resources, Transportation Services, Housing Services, Substance Use Services, Domestic Violence Support, Mental Health Resources, Legal Aid, COVID Services, Social/Community Support, Food Pantry, Insurance Navigation, or Immigration Services.

d STI: sexually transmitted infection.

e Data not collected.

#### Intervention Satisfaction

The postsession satisfaction mean across sessions was 6.23 (SD 1.09) on a 7-point scale with a Cronbach α of 0.85. Satisfaction for the interventionist was high, with 56% (31/55) of participants indicating they were very satisfied and 29% (16/55) indicating they were mostly satisfied. While a majority remained engaged and satisfied, about 9% (5/55) of participants were indifferent or mildly dissatisfied.

### Platform Use and Satisfaction

Overall, intervention participants had higher mean total logins and mean total minutes in the PrEPresent app than control participants. The mean total logins on the platform was 6.31 (SD 9.27) in the control arm and 15.93 (SD 15.85) in the intervention arm (95% CI 5.01-13.58; Table 4). A similar trend was observed in the mean total minutes spent on the platform, with control participants spending 3.03 (SD 5.70) minutes in the app on average compared to 9.51 (SD 11.47) minutes in the app in the intervention arm (95% CI 3.51-9.51). Platform satisfaction was also rated more highly among intervention participants, with 41% (23/56) stating they were very satisfied compared to 26% (15/58) of control participants. More than two-thirds (34/56) of intervention participants stated PrEPresent helped them deal with the challenges they have faced getting on PrEP compared to 43% (25/58) of control participants (Table 4). Satisfaction around the app platform was universally high across both RCT arms, though overall use of the platform was low. Around 70% (81/114) of participants agreed or strongly agreed that the platform was easy to use. At least 70% (82/114) of participants in both arms stated PrEPresent helped them make healthier choices at a 4 or above on a 0‐7-point sliding scale. There were high ratings on levels of trust for the information presented in the app, with no intervention participants disagreeing or strongly disagreeing that they trust the information in PrEPresent. Most participants would use the app in the future.

**Table 4.** The 3-month mHealth^a^ platform engagement and usability measurements.

Variables	Total control (n=75)	Total intervention (n=72)	3-month control (n=58)	3-month intervention (n=56)
Platform use				
Total logins				
Mean (SD)	6.31 (9.27)	15.93 (15.85)	—^b^	—
Median (IQR)	2.00 (1.00-7.50)	11.00 (4.00-22.25)	—	—
Total minutes				
Mean (SD)	3.03 (5.70)	9.51 (11.47)	—	—
Median (IQR)	0.86 (0.04-2.73)	5.30 (2.01-13.06)	—	—
Overall, how satisfied are you with PrEPresent?, n (%)				
Very satisfied	—	—	15 (26)	23 (41)
Somewhat satisfied	—	—	30 (52)	22 (39)
Somewhat unsatisfied	—	—	12 (21)	10 (18)
Not satisfied	—	—	1 (2)	1 (2)
Has PrEPresent helped you deal with the challenges you’ve faced getting on PrEP^c^?, n (%)				
Decline to answer	—	—	20 (35)	13 (23)
No, it really didn’t help	—	—	13 (22)	9 (16)
Yes, it helped	—	—	17 (29)	28 (50)
Yes, it helped a great deal	—	—	8 (14)	6 (11)
PrEPresent was easy to use, n (%)				
Strongly disagree	—	—	0 (0)	2 (4)
Disagree	—	—	4 (7)	5 (9)
Undecided	—	—	13 (22)	9 (16)
Agree	—	—	24 (41)	23 (41)
Strongly agree	—	—	17 (29)	17 (30)
PrEPresent was hard to use, n (%)				
Strongly disagree	—	—	15 (26)	12 (21)
Disagree	—	—	36 (62)	35 (63)
Agree	—	—	5 (9)	7 (13)
Strongly agree	—	—	2 (3)	2 (4)
I trust the information in PrEPresent				
Strongly disagree	—	—	1 (2)	0 (0)
Disagree	—	—	1 (2)	0 (0)
Agree	—	—	26 (45)	26 (46)
Strongly agree	—	—	30 (52)	30 (54)
I would use PrEPresent in the future if it were available, n (%)				
Strongly disagree	—	—	3 (5)	6 (11)
Disagree	—	—	14 (24)	7 (13)
Agree	—	—	34 (59)	32 (57)
Strongly agree	—	—	7 (12)	11 (20)
PrEPresent helps me make healthier choices in my life^d^, n (%)				
0‐3	—	—	15 (26)	17 (30)
4‐7	—	—	43 (75)	39 (70)

a mHealth: mobile health.

b Data not available.

c PrEP: pre-exposure prophylaxis.

d 0-7-point sliding scale.

## Discussion

### Principal Findings

Overall, within a sample of racially and ethnically diverse SGM youth, PrEPresent met predetermined objectives for feasibility and acceptability in the pilot RCT portion of the research. The project did not see high rates of PrEP uptake when compared to other PrEP uptake interventions [29-34] or when compared against documented PrEP use within similar cohorts outside of intervention settings [11,35]. Additionally, use of the mHealth platform was overall low across both study arms compared to other iterations of the HMP platform [24,36-38]. The outcomes of this project are valuable in understanding how future projects should engage SGM youth in HIV prevention efforts, particularly using health technology.

### PrEP Uptake and Adherence

Although PrEP uptake within the intervention arm was 25% (14/56) and 19% (11/58) in the control arm, these are lower uptake percentages compared to other interventions assessing PrEP initiation [30-32]. A few studies have noted intervention-level PrEP uptake levels similar to PrEPresent but with a greater difference between control and intervention arms [29,34]. The outcomes from PrEPresent add to previous literature demonstrating the mixed success that PrEP uptake and adherence projects have had in activating SGM youth in the PrEP care cascade. In the TechStep project, conducted across 5 major US cities, there was no difference between intervention arms (text plus, text only, web app plus, and web app only) and control arms in PrEP uptake within 254 transgender and gender-expansive youth [39]. In a mobile messaging mHealth app study for 1226 SMM, high-risk HIV-negative groups had a higher odds of HIV testing and PrEP use, but these behavioral changes were not noted for the participants with low-risk HIV-negative groups [33]. In the theory-driven MyChoices app for cisgender young sexual minority men, HIV testing was higher within the intervention arm, but no difference in PrEP uptake was noted [40]. High acceptability across these projects indicates that while SGM youth find mHealth interventions usable, more work needs to be done to understand how to translate app engagement to behavior change. Future mHealth projects targeting PrEP uptake powered for efficacy are required to understand how mHealth can help SGM youth overcome barriers they face to starting PrEP.

Although adherence was not an outcome for this project, we used the UrSure test as an indicator of PrEP uptake in conjunction with self-reported survey data. Biological markers of PrEP use continue to be important in conjunction with self-reported PrEP use, as evidenced in both clinical and research settings [41,42]. In a study assessing the feasibility of urine testing for PrEP adherence in a sample of young people, it was found to be theoretically more acceptable than phlebotomy draw, finger prick, or hair test [43]. Despite this, completion of the at-home UrSure urine test for PrEP adherence in this study was low across both arms. Of those who completed the 3-month surveys, only around 60% (69/114) also completed the UrSure testing, accounting for 47% (69/147) of the total initial sample from baseline. The study team noted several challenges in biological result collection. Due to privacy concerns and short follow-up windows for specimen delivery, study team members required participant verification of address before sending test kits. Future projects may consider automating check-in processes for verifying participant addresses before their testing window opens or obtaining consent to send materials without verification. Many participants received test kits but did not complete them. Increasing the specimen completion incentive instead of prioritizing a bonus incentive may have increased test kit return.

It is possible that, due to low PrEP uptake in our cohort, participants were not as interested in understanding their PrEP adherence levels. In a 2023 qualitative feedback study on self-collected STI testing from a similar participant base, low levels of return were noted for those who had less concerns about a positive HIV and STI diagnosis [44]. Additional language about the importance of test kit completion to the study aims, regardless of PrEP uptake, may have helped increase biomarker return. Postintervention qualitative feedback with PrEPresent participants around PrEP adherence testing may provide insights into motivators and barriers to test completion. Biological markers of adherence delivered key insights into PrEP uptake across the cohort, and future studies may consider pilot testing adherence testing protocols with members of YABs to understand acceptability and to troubleshoot sample collection.

Rates of adherence to the UrSure tests were also low among our participants. The UrSure point-of-care test measures adherence in the last 48 hours. While positive adherence results on these biological markers can deliver important PrEP uptake information, it is difficult to interpret levels of protection against HIV based on these results. It has been established that 4 (or more) doses of oral PrEP per week over time create adequate protection in rectal tissues [45], whereas 6‐7 doses of oral PrEP per week are needed to create adequate protection in vaginal tissues [46]. Participants could have protective levels of PrEP in their system but test nonadherent on the UrSure point of care test, depending on when pills were taken. As noted in a review of PrEP adherence testing methods, understanding sexual activity type and sex assigned at birth is important in contextualizing urine adherence results, particularly those that measure dosage in the last 48 hours [47]. Additionally, though rates of injectable PrEP were low in our cohort, injectable Cabotegravir is not detected by the UrSure test. Future research and clinical settings using adherence measures must note that evolving PrEP landscapes and the use of on-demand PrEP may impact results regarding adherence across different forms of testing.

### Intervention Feasibility and Acceptability

A total of 50% (36/72)of participants attended a second intervention session, meeting the metric for feasibility. Completion of the fourth closing session was low, at only 5 (7%) participants. Four intervention sessions, spaced every 4 weeks, were offered to participants within the 3-month active intervention period. Scheduling this number of sessions may not have seemed practical for our participant population. Referrals dropped in the third and fourth sessions, possibly indicating that a reduced number of sessions may still meet participant needs [32,34,48]. An initial session delivering PrEP information to SGM youth may be supportive in their decision to pursue a PrEP prescription, as motivation and PrEP knowledge were the most discussed intervention session topics during the first session. Postsession satisfaction across sessions remained high and scored well above our target average of 4 for feasibility. Overall satisfaction with the PrEPresentative was also high, with the majority of participants rating their satisfaction as “very satisfied.” These results demonstrate that in a population of SGM youth, a peer-support navigation intervention for PrEP uptake is acceptable and feasible.

Delivery of the intervention sessions varied across participants and sessions. YAB feedback dictated offering a variety of session modalities because of the overall increase in video meetings throughout the COVID-19 pandemic. Future interventions should use community feedback to understand desires for intervention delivery. One study citing clinical strategies to adjust to pandemic-constrained PrEP access noted that newly offered telehealth access for services remained in use even after COVID-19 cases had stabilized [49]. An overview of PrEP access via telehealth published in 2022 found both publicly funded clinical settings and private companies using telehealth for PrEP delivery and highlighted the use of a California state-funded tele-PrEP delivery program [50]. Given that the study was deployed in the post-COVID-19 pandemic period in a state offering digital PrEP access, the novelty of an app-delivered peer support navigator for PrEP uptake may have been less appealing during this time.

One reason the acceptability and feasibility of the intervention may have been high despite low platform use is that PrEPresent prioritized community-based collaboration throughout all phases of the project. App feedback was overwhelmingly positive, with most participants trusting the information in the app and agreeing that it helped them make better choices. Youth working groups and YABs comprised of racially and ethnically diverse SGM youth engaged with the project to understand the similarities and differences between young sexual minority men and transgender and nonbinary populations and how a mHealth intervention could best serve them both. Members of the research team, from research assistants to the interventionist, represent different components of the community represented within this sample. Uplifting community voices should continue to be prioritized in prevention strategies to meet community members’ needs.

### Platform Use and Satisfaction

Use of the PrEPresent intervention was overall low compared to other iterations of mHealth projects built on the HMP Platform [23,24,36]. Primary login to the app was verified for all participants. Intervention participants did have similar login rates to the app as other mHealth platforms [24,38], which may be due to using scheduling features to meet with the interventionist. Most intervention participants chose to engage with the PrEPresentative outside of the app (through phone call or video call), but this alone does not fully explain the low time spent within the app. The project team worked to adapt the platform to drive digital interaction, but certain features shown to increase engagement in other mHealth interventions were not integrated. Features such as use-based points or digital currency [36], app-based health monitoring and tailored messaging [22,51], serious gaming or enhanced gamification [52,53], peer mentor engagement [54], and access to HIV prevention materials such as home tests or condoms and lube [23,51] have been demonstrated to engage similar participant populations in mHealth studies. Reviews of published mHealth findings have found links between higher app use and behavior change [55]. mHealth interventions hoping to combine app-based features with external features such as two-way SMS text messaging or peer health navigators may consider additional in-app incentives to encourage user engagement and should prioritize the collection of app paradata to explore participant use.

### Limitations

Several limitations of this study should be noted. Randomization for the pilot RCT was conducted on a 1:1 basis with no stratification, and as a result, some imbalances may have occurred. While this is an important consideration, it is not unique to this study and reflects a common limitation in randomized trials with modest sample sizes. Additionally, PrEPresent was not powered to look at comparisons between subgroups.

This study population was drawn from the Los Angeles metropolitan area, and it reflects the demographic profile in Los Angeles. While we did recruit a racially diverse sample, we cannot compare outcomes within different racial or ethnic groups or generalize results to these groups. Because recruitment for the project took place in a large-scale US city, results may not be generalizable to other geographic areas where factors impacting PrEP uptake may differ, such as access to insurance coverage or clinical spaces specializing in PrEP services. In addition, a subset of participants was recruited from a previous observational cohort study focused on HIV prevention with SGM youth of color. While participants had not received any digital health intervention or app-based support related to PrEP during their involvement, our sample size constrained our ability to conduct subgroup analysis to understand potential impacts on intervention engagement. Future studies involving existing cohorts may include prior participation as a stratification variable or covariate where appropriate.

Additionally, while we intended to include the validated System Usability Scale (SUS) in our survey instrument, we discovered during data analysis that supplementary usability questions were mistakenly identified as the SUS in the survey documentation. As a result, we were unable to calculate SUS scores using the validated measure. Given this error, we used an alternative breakdown of usability constructs as presented in Table 4, which aligns with the domains outlined in our grant proposal and captures key facets of user experience (eg, engagement, ease of use, and satisfaction). Although not based on the SUS, this adapted usability assessment still provides valuable insights into intervention performance and supports the interpretability of our findings. Future mHealth work with this population should further psychometric evaluation of existing validated measures while also prioritizing the development of tools that reflect the lived experiences of diverse young populations at risk for HIV.

Finally, attrition by 6 months was higher than anticipated, leading to reduced power and potential bias in outcome estimates measured at that time point. As such, we felt it most appropriate to focus on the more complete and rigorously collected 3-month data for this initial report, as including 6-month outcomes would not have strengthened the interpretation of the primary study aims. We are currently exploring the 6-month data in secondary analyses and plan to report those findings—particularly related to longer-term patterns of engagement and behavioral trajectories—in a subsequent paper.

### Future Directions

As demonstrated in the outcomes of this project, one limitation of many mHealth interventions is engagement in the app or web-based tool [55]. In a similar mHealth study, while community events, group sessions, access to services, and use of their mHealth app were all used, app use averaged about 3.35 minutes per user, and PrEP outcomes were not significant [56]. Understanding the best features to drive long-term use and interest in app platforms and to track and analyze the use of those features will move app-based PrEP interventions toward higher rates of use, which may lead to better prevention outcomes. Engagement linked to features available through mHealth platforms will also need to consider scalability (the ability to offer the app and features to larger amounts of people) in terms of translational efforts in being integrated into clinical care [57,58]. Future studies will need to prioritize the components of their interventions that work best within their participant population, such as interventionist visits in this study, to understand how to guide engaging mHealth projects into translatable tools for HIV prevention.

Throughout the course of this project’s development, changes in the types of PrEP available from foundational research to pilot RCT execution occurred. Future mHealth apps supporting PrEP uptake and adherence will need to be developed within the evolving context of PrEP modalities used among SGM communities [59,60]. Across the United States, injectable PrEP use alone increased from 1.1% to 2.5% of all PrEP prescriptions from 2022 to 2023 [61]. Recently, a longer-acting injectable PrEP option delivered in-clinic twice-yearly, Lenacapavir, was approved for all adolescents and adults [62]. Injectable PrEP may have a lower barrier to uptake and adherence, particularly for those accessing the medication through Medicare coverage, as a new National Coverage Determination expanded coverage to both oral and injectable PrEP medications with no cost-sharing [63]. With the introduction of injectable PrEP and the popularity of on-demand PrEP, daily PrEP adherence may not be as relevant of a metric for HIV prevention moving forward. Instead, initiation, persistence, and clinical appointment attendance may need to be addressed through innovative app features.

### Conclusions

Results from the PrEPresent study indicate that SGM youth at a greater likelihood of HIV acquisition are willing to engage with a peer-support navigator through a mHealth platform to understand more about PrEP and PrEP uptake. Access to this type of mHealth app in combination with peer navigation may increase PrEP uptake in individuals outside the PrEP care continuum. Among those in the intervention arm, the app and PrEPresentative were satisfactory, although overall engagement was low. Additional mHealth studies around prevention should continue to include and elevate the voices of their participant populations through community-engagement processes to ensure they meet the needs of SGM people.

## Supplementary material

10.2196/69255 Checklist 1 CONSORT e-Health Checklist (V1.6.1)

## Abbreviations:

HMP: HealthMpowerment
mHealth: mobile health
PrEP: pre-exposure prophylaxis
RCT: randomized control trial
SGM: sexual and gender minority
STI: sexually transmitted infection
SUS: System Usability Scale
YAB: Youth Advisory Board
